# The Effect of Behaviour and Diet on the Rumen Temperature of Holstein Bulls

**DOI:** 10.3390/ani9111000

**Published:** 2019-11-19

**Authors:** Naomi H. Rutherford, Alan W. Gordon, Francis O. Lively, Gareth Arnott

**Affiliations:** 1 Agri-Food and Biosciences Institute, Large Park, Hillsborough BT26 6DR, UK; francis.lively@afbini.gov.uk; 2 School of Biological Sciences, Queens University Belfast, Belfast BT9 7BL, UK; garnott@qub.ac.uk; 3 Agri-Food and Biosciences Institute, 18a Newforge Lane, Belfast BT9 5PX, UK; alan.gordon@afbini.gov.uk

**Keywords:** rumen temperature bolus, drinking, concentrates, behaviour, agonistic

## Abstract

**Simple Summary:**

The use of precision technology within agriculture is growing rapidly. Rumen temperature boluses are primarily used for the detection of ill health, but also have uses in detecting estrus and the onset of parturition. Research has shown that water intake and diet can impact rumen temperature. However, little emphasis has been placed on the impact of behaviour, particularly agonistic interactions, which are common amongst young bulls. In fact, there is a clear knowledge gap surrounding the effect behaviour has on physiology, particularly core body temperature. Thus, the aim of this study is to investigate the impact of behaviour and diet on the rumen temperature of Holstein bulls, both at grass, and in a housed environment. The results from this study indicate that although significant differences in rumen temperature exist between behaviour groups, these rumen temperatures are all within the normal temperature range. Therefore, behaviour should not impact the accuracy of the detection of ill health. Furthermore, diet had no effect on rumen temperature.

**Abstract:**

Rumen temperature boluses are becoming increasingly used as a means of monitoring core body temperature for the detection of ill health. However, the effect of behavior on rumen temperature is largely unknown. This research investigates the impact of behaviour and diet on the rumen temperature of Holstein bulls, both at grass, and in a housed environment. Rumen temperature was recorded at five-minute intervals using a bolus. Direct observations were conducted on young bulls in two studies (i) at grass (*n* = 30) and (ii) while housed (*n* = 32). In addition, activity monitors were attached to bulls at grass (*n* = 24). Within each study, diet differed by the level of concentrate supplementation. There was no effect of diet on rumen temperature. Significant differences in rumen temperature were observed between behaviour groups for bulls at grass (*p* < 0.001) and housed (*p* < 0.001). Furthermore, drinking resulted in the lowest rumen temperature (grass 35.97 °C; housed 36.70 °C). Therefore, rumen temperature is affected by behavior; however, the temperatures recorded were not outside the normal temperature range for healthy cattle.

## 1. Introduction

Core body temperature is often measured via rectal temperature [1]; however, this method involves regular handling and has practical limitations [2]. Furthermore, fluctuations in temperature may be missed due to the sampling procedure [3]. Rumen temperature boluses are a novel technology which allows for continuous, non-invasive monitoring [4,5]. Commercially they have a number of uses, including the detection of ill health [4], estrus [6], and the onset of parturition [7]. Heat is produced in the rumen due to fermentation and the activity of the microbiome [8]. As a result, rumen temperature has been reported to remain 0.57 °C greater than rectal temperature [4]. However, variations in rumen temperature could be expected due to diet. Concentrates are easily fermentable [9], and thus, a high proportion in the diet could lead to increased heat production in comparison to a forage-based diet. The effect of drinking on rumen temperature has been investigated and is reported to cause a rapid decline. The extent and duration of which depends largely on the temperature and volume of water consumed [10,11].

However, while factors such as ill-health, oestrus, parturition, and drinking are well known to influence rumen temperature in predictable ways, the influence of behavioural activities remains to be explored. Cattle exhibit three main behaviours: feeding, ruminating, and resting, which have been reported to account for up to 95% of the animals’ time. However, for the remainder of the time, cattle may be engaged in an extensive range of behaviours [12]. Cattle are social animals [12,13] who will interact with one another primarily through agonistic (fighting, head to head pushing, and butting), affiliative (grooming), and sexual (mounting and flehmen response) behaviours [14]. Agonistic behaviours are often associated with competition for space or resources (resting space, access to feeding space), particularly under intensive production systems [15]. In addition, these behaviours enable the formation of a social hierarchy within a group [16]. Sexual behaviours, particularly mounting between bulls, can also play a role in establishing dominance relationships [17] while affiliative behaviours are associated with the development of social bonds [18], together with having coat hygiene benefits [19].

Behavioural activities associated with increased physical activity or stress will lead to increased heart rates [20,21] and body temperatures [5,6,7,8,9,10,11,12,13,14,15,16,17,18,19,20,21,22,23]. During this time, blood is redirected to vital organs and muscles in order to meet the additional metabolic requirements [24]. A short term rise in core body temperature, known as hyperthermia, has also been suggested to be caused by agonistic behaviour. For example, Timsit et al. [4] investigated the use of rumen temperature boluses for the early detection of bovine respiratory disease (BRD) in young bulls, reporting that 27% of rumen hyperthermia cases were not followed by clinical signs of BRD. The author speculated that these were caused by agonistic behaviour rather than ill-health. Agonistic interactions are often magnified following mixing or re-grouping, which is associated with establishing a new dominance hierarchy. This manipulation of the social environment leads to an increased occurrence and intensity of agonistic interactions, particularly if animals are of a homogenous weight [25]. Physiological changes, such as an increase in lactate, plasma cortisol, and creatine kinase, are also associated with mixing [26].

Temperament is a measure of an animal’s behavioural response to standardised environmental or social stimuli [27,28,29]. Fearfulness and aggressiveness are two of the key traits that are used to define an animal’s temperament [28]. Temperament has been shown to impact animal performance in terms of immune function, growth rates, and carcass characteristics [30,31,32]. In addition, cattle with different temperaments have been shown to exhibit physiological differences. For instance, temperamental (or excitable) cattle have greater basal body temperatures than those considered to be calm [33,34]. The same goes for stress hormones; Burdick et al. [35] reported that temperamental bulls had significantly greater basal levels of cortisol and epinephrine than calm bulls. Similar findings have been documented in relation to dominance; bulls with a low or high social rank had a significantly greater plasma cortisol and neutrophil to lymphocyte ratio than bulls of a medium social rank [36].

However, the assessment of physiological changes associated with behaviours during mixing or temperament classification is based on a small number of time points with behaviours being grouped over a long duration. Thus, the relationship between specific behavioural activities and animal physiology is poorly understood, particularly within a stable social environment. This is an important knowledge gap to address, given the increasing commercial uptake of rumen temperature boluses in cattle. At present, the extent to which behavioural activity could act as an important confounding factor [4] remains to be explored.

Thus, the objective of this study is to investigate the impact of behaviour, particularly agonistic interactions on rumen temperature of young bulls. Furthermore, the current study also investigates the role of diet as a source of variation in rumen temperature. Specifically, we hypothesise that behaviour involving relatively high levels of physical activity (e.g., agonistic interactions), and a high concentrate diet, will be associated with increases in rumen temperature.

## 2. Materials and Methods

This study was undertaken from July to October 2017 at the Agri-Food and Biosciences Institute (AFBI), Hillsborough, Northern Ireland. AFBI is located at latitudes and longitudes of 54.45° and −6.07°, respectively, and is 91 m above sea level. The area has a mean annual temperature of 9.5 °C and a mean annual rainfall of 902 mm. All experimental procedures were conducted in compliance with the United Kingdom Animals (Scientific Procedures) Act 1986. This study consisted of two data collection periods, one comprising 30 bulls at grass (24 of these also had IceQubes fitted for activity monitoring), and the second consisting of 32 bulls in a housed environment.

### 2.1. Study one: Bulls at Grass

#### 2.1.1. Animals and Rumen Temperature

**Figure 1:**
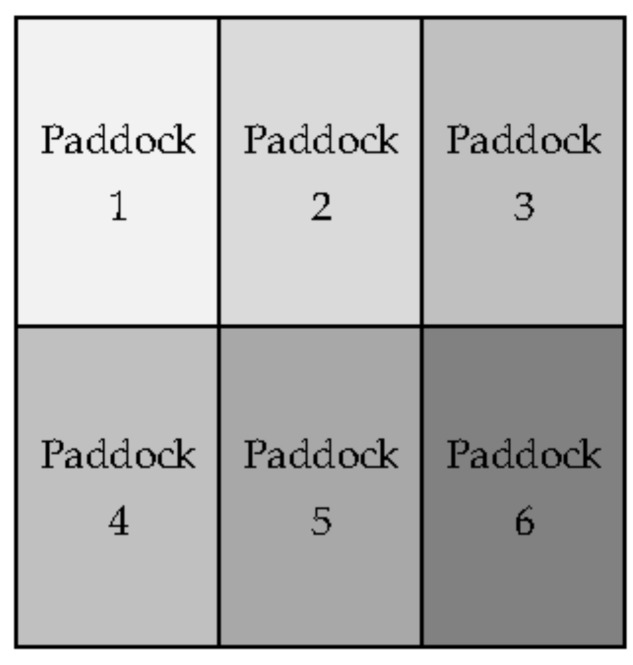
Paddock set up in Study 1.

A total of 84 bulls were managed in six groups of 14 in a four paddock rotational grazing system. The paddock set up consisted of four blocks of six paddocks, with all six groups being grazed within one block of paddocks at a time (Figure 1). The boundaries of the paddocks were determined by electric fencing. As part of a wider production study, the six groups were offered one of three summer diets; (i) grazed grass only, (ii) grazed grass with 2 kg concentrate supplementation, and (iii) grazed grass with ad libitum access to concentrates. The chemical composition of grass was determined via NIRS analysis, and that of concentrates was determined by wet chemistry analysis.

Each bull had a rumen temperature bolus (Thermobolous small, Medria, Châteaubourg, France) administered at three months of age. Each bolus had a battery life of three years. The factory-calibrated bolus recorded rumen temperature to a tenth of a degree Celsius every five minutes [4,5]. A radio base was placed in the centre of each block of paddocks, to allow the data from each bolus to be automatically downloaded.

A weather station (Davis Vantage Pro 2, Davis Instruments, Hayward, CA, USA) situated centrally within the farm was used to record external ambient temperature (°C) and relative humidity (%) at 30 m intervals. Temperature humidity index (THI) was calculated from the data collected using the formula below where T is ambient temperature (°C), and RH is relative humidity expressed as a proportion [37].

THI = 0.8T + RH (T ‒ 14.4) + 46.4

#### 2.1.2. Observations

A sub-sample of these bulls (*n* = 30) (196 ± 5.3 days of age and 195 ± 8.9 kg live weight) were selected based on age and balanced for paddock and diet. Bulls were identified using coloured collars. Direct observations were conducted by one trained individual, with a total of 10 observation days completed over a period of 24 days in July and August 2017. Each observation period lasted six h (1000 h to 1600 h), giving a total of 60 h of direct observations per animal. The behaviours recorded are shown in Table 1. Behaviour sampling was continuous, with the start time of each behaviour being recorded; thus, the duration of a behaviour was taken as the interval between two behaviours. Each behaviour was then assigned to a behaviour group, which categorised similar behaviours together. To allow appending with rumen temperature data, behaviours were condensed so that one behaviour group was allocated to each five-minute interval. Where more than one behaviour group occurred within each interval, behaviours were selected based on the rank shown in Table 1. This ranking was designed so that behaviours that were of a particular interest within this study were selected. Drinking was selected as the highest ranking behaviour as it is the only factor that is currently well-validated to cause a decline in rumen temperature [10,11]. The incidence rate (IR, %) for each behaviour group was calculated on an hourly basis, providing a quantitative measure of the proportion of time spent on each behaviour.

#### 2.1.3. Activity Monitoring

IceQubes (IceRobotics, Edinburgh, Scotland, UK) were fitted to 24 randomly selected bulls (four bulls per paddock) for a period of 30 days while bulls were at grass. IceQubes were attached to the right hind leg, as reported by Finney et al. [38], and recorded standing durations, lying duration, lying bouts, steps, and a motion index. IceQubes recorded data at 15 m intervals; thus, the corresponding rumen temperature was the mean of the three temperatures recorded within each interval. Two IceQubes (one bull grazed with 2 kg concentrates, and one bull grazed with ad libitum concentrates) were dislodged during the study and thus fell off, leaving 22 bulls with an IceQube for the full study period.

### 2.2. Study Two: Bulls Housed

#### 2.2.1. Animals and Rumen Temperature

The bulls used in Study 1 were housed in October 2017 to commence their finishing period. Each group of 14 was split according to live weight into three pens of four bulls and one pen of two bulls. All pens were of the same dimensions (3.4 × 2.7 m) and had slatted floors. A total of 32 bulls (256 ± 3.9 days of age and 275 ± 7.4 kg live weight) across eight pens were observed during Study 2; only those that were penned in groups of four were observed. These bulls were balanced over four dietary treatments (eight bulls per diet) and were offered ad libitum grass silage with varying levels of concentrates. The bulls that had been offered 0 and 2 kg of concentrates at grass, both had their concentrate allowance increased by 2 kg/h/d and thus were on 2 and 4 kg of concentrates, respectively. The bulls on ad libitum concentrates at grass, were maintained at this concentrate level. A fourth dietary group was introduced to this study; these bulls had spent the summer housed with access to ad libitum grass silage and concentrates. The chemical composition of the grass silage and concentrates were determined using a wet chemistry analysis.

Bulls had a rumen temperature bolus administered as per the animals in Study 1 (Section 2.1.1). A radio base was placed centrally within the shed, to download data automatically. Ambient conditions were recorded using two iButtons (Hydrochron, DS1923 F5, Maxim Integrated, Hayward, CA, USA) placed at either end of the finishing house. iButtons were secured to the bars of the pen just above animal height, ensuring the face of the iButton was not obscured. Ambient temperature (°C) and relative humidity (%) were recorded every 10 m. THI was calculated using the formula outlined in Section 2.1.1.

#### 2.2.2. Observations

Prior to observations commencing, bulls had an acclimatisation period of 6 days after housing. Video footage of each pen was recorded, and bulls were identified using coloured collars. Videos were later scored by one trained individual using Observer XT 13 software. Observations were conducted on alternate days (*n* = 7), with four one-hour observations completed per day commencing at 03:59, 09:39, 14:10, and 20:43. Thus, a total of 28 h of observations were completed per animal. Within each one-hour observation period, the bulls were scored continuously using the ethogram shown in Table 1. Individual behaviours were assigned to a behaviour group, and the method of ranking behaviours, and calculating incidence rates outlined in Section 2.1.2 was applied.

### 2.3. Statistical Analysis

All statistical analyses were conducted using Genstat (19th edition). Summary statistics were conducted on THI and feed composition, with mean and SE values being reported. Rumen temperature was examined using linear mixed model (LMM) methodology, and the REML estimation method. For Study 1, behaviour group and diet were fitted as fixed effects while animal ID was fitted as a random effect in the modelling process. Study 2 was analysed using the same model with the addition of the pen number being included as a random effect. Summary statistics were completed on the incidence rate of behaviour groups during each study. The effect of diet on behaviour incidence rate was analysed as an LMM with animal ID as the random effect for Study 1; and animal ID and pen number as random effects in Study 2. In all cases, if any effect was significant then pairwise differences among treatment levels of the effect were assessed using Fisher’s least significant difference test. To assess the relationship between rumen temperature and step count, and whether this differed depending on the production system, linear mixed model methodology using the REML estimation method was used. The animal was fitted as a random effect, while step count, production system, and their interaction were fitted as fixed effects in the modelling process. If there were significant relationships established then pairwise differences between the slopes of each model were assessed using a *t*-test. In all cases, data was not transformed, and the adequacy of the model fits was assessed by visual inspection of residual plots.

## 3. Results

Mean THI was 56.88 ± 0.128 and 56.25 ± 0.096 during Study 1 and Study 2, respectively. The chemical composition of the feed offered during both studies is shown in Table 2. Mean rumen temperature in Study 1 was 38.09 ± 0.094 °C, while that of Study Two was 38.59 ± 0.037 °C. Diet had no significant effect on mean rumen temperature in either of the two studies, and thus, the results are not shown.

**Figure 2:**
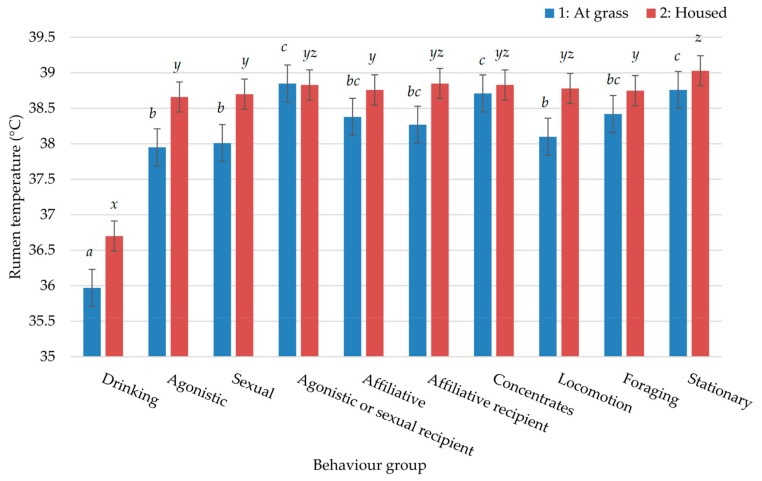
Effect of behaviour on rumen temperature during the two studies. a, b, c represent significant differences (*p* < 0.001) between behaviour groups in Study 1: at grass. x, y, z represent significant differences (*p* < 0.001) between behaviour groups in Study 2: housed.

**Figure 3:**
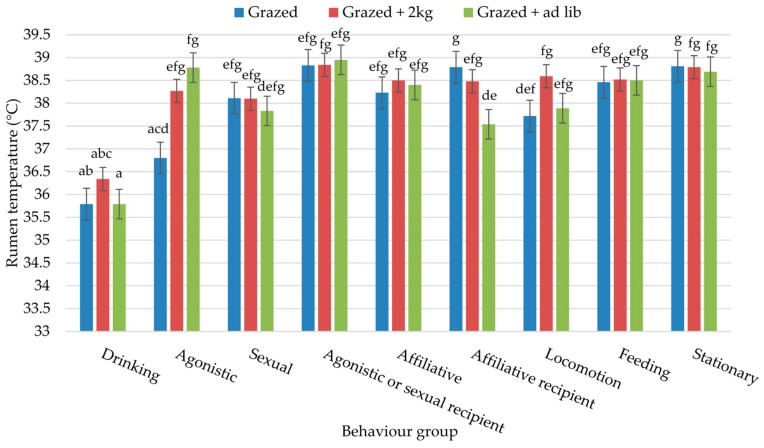
Interaction between behaviour and diet for bulls on Study 1: at grass; a–g represent significant differences (*p* = 0.047) between behaviour groups.

Figure 2 outlines the effect of each of the ten behaviour groups on rumen temperature during the two study periods. In both instances, as expected, drinking caused a significant decline in rumen temperature, with a mean of 35.97 and 36.70 °C while at grass and housed, respectively. Interactions with other bulls resulted in similar rumen temperatures in both studies, with only agonistic or sexual recipients in Study 1 displaying a significant elevation in comparison to that of agonistic and sexual behaviours. A mildly significant interaction (*p* = 0.047) between behaviour and diet was observed in Study 1 (Figure 3). Drinking had a consistently lower rumen temperature for bulls on all three diets. Agonistic behaviours for grazed bulls proved to be the only group that had a similar rumen temperature to that of drinking. Across the remaining behavior groups, there were no consistent patterns of interactive effects across this marginally significant interaction. There was no significant interaction for Study 2; therefore, the results are not presented.

Table 3 shows that in both studies, the most common behaviour group recorded was stationary. In Study 1, bulls that were grazed with ad libitum concentrates had the greatest incidence rate of stationary behaviours (Table 4) and the lowest incidence of feeding. In contrast, Study 2 showed no significant difference in stationary behaviour between diets. Housed + ad lib bulls displayed the greatest incidence of affiliative behaviours; while grazed and grazed + 2 kg bulls spent the greatest time consuming forage.

**Figure 4:**
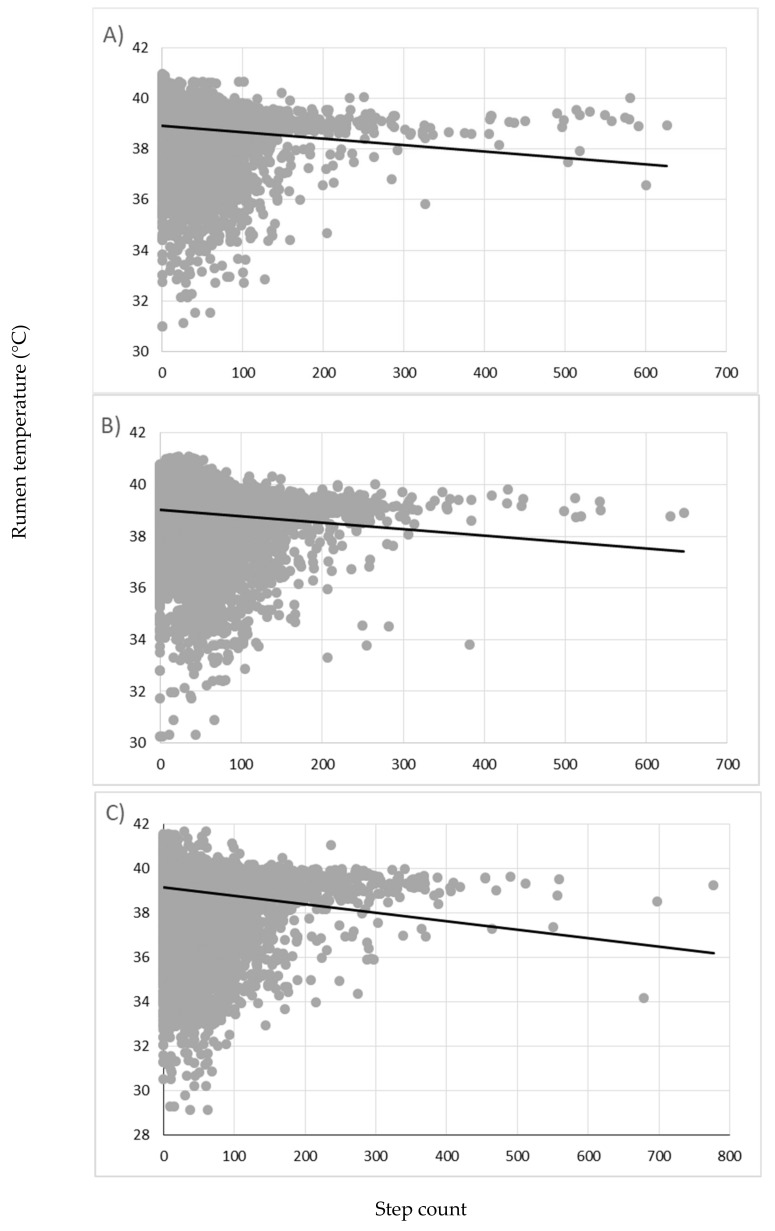
The effect of step count on the rumen temperature of bulls on three different diets; (**A**) grazed only, (**B**) grazed with 2 kg concentrate supplementation, and (**C**) grazed with ad libitum access to concentrates. (**A**) y = ‒0.002534χ + 38.85 ^a^ (**B**) y = ‒0.002456χ + 38.99 ^a^ (**C**) y = ‒0.004077χ + 39.06 ^b^. a, b represent significant differences between the interaction of step count and diet (*p* < 0.001).

There was a negative relationship (*p* < 0.001) between step count and rumen temperature. Furthermore, Figure 4 shows there was a significant interaction between step count and diet, with bulls that were grazed with ad libitum access to concentrates displaying a significantly more negative relationship.

## 4. Discussion

Heat stress in dairy cattle has been shown to cause a rise in rumen temperature [39]. However, mean ambient conditions observed in this study were considerably below the THI threshold for heat stress of 65 outlined by Ammer et al. [39]. Therefore, the bulls in this study were not under an excessive heat load, and rumen temperature is unlikely to have been affected by ambient conditions.

The mean rumen temperature from Study 1 and Study 2 is in agreement with that of AlZahal et al. [40], but lower than that observed by Bewley et al. [8]. Although there was a marginally significant interaction between behaviour and diet in Study 1, there was no consistent effect of diet on the mean rumen temperature during the observational period. This is in agreement with the findings of Petzold et al. [41] and Castro-Costa et al. [42] who offered high and low concentrate diets to periparturient dairy cows and non-lactating goats, respectively. In contrast, other studies involving lactating [40] and non-lactating dairy cows [43] have found that high concentrate diets result in a greater rumen temperature, with a reduced rumen pH also being observed. This negative relationship between rumen temperature and pH is characteristic of cattle undergoing a subacute ruminal acidosis challenge [40], the intensity of which is compounded if the rumen environment is not sufficiently adapted to concentrate feeding [9]. Therefore, as rumen temperature was consistent across the diets in this study, it is unlikely that the bulls were in an acidotic state.

Figure 2 indicates that concentrate feeding did, however, cause an immediate rise in rumen temperature. Concentrates contain high proportions of easily fermentable carbohydrates [9,43] and therefore are much quicker to ferment in the rumen than forage-based feeds. Furthermore, Tafaj et al. [44] reported that fermentation was more intense within the top and middle layers of digesta in the rumen. Thus, as recently ingested feed is located at the top of the rumen, fermentation occurs imminently [44,45]. Therefore, heat, a by-product of fermentation, is generated soon after the ingestion of concentrates, hence justifying the rise in rumen temperature observed in this study. Feeding incidence was greatest for bulls on a grazed diet with no concentrate supplementation (Table 4). Forage-based diets result in a slower intake rate in comparison to concentrate based diets [46]. Thus, greater bouts of feeding would be expected in order to fulfill intakes.

Figure 2 and Figure 3 show no significant difference between being the donor or recipient of affiliative behaviour in terms of rumen temperature. In addition, rumen temperature during these behaviours was not significantly different from agonistic or sexual interactions (with the exception of agonistic behaviour of grazed bulls (Figure 3) which will be addressed later), thus indicating that it is difficult to differentiate between behavioural interactions based purely on rumen temperature. The greater incidence of affiliative behaviours for housed ad lib bulls in Study 2 may be reflective of tighter social bonds. These bulls were also housed as a group of 14 during the summer when the other three dietary groups were at grass; and, therefore, had spent longer in a housed environment. Confinement systems are known to lead to increased stress levels [47], while affiliative behaviours often act as a displacement activity [48]. Furthermore, affiliative behaviours have been shown to cause a calming effect for the receiver, identified by a decline in heart rate in dairy cows [49].

As the two studies were not conducted at the same time, a direct comparison cannot be made between behavioural incidences rates. However, combining the five behaviour groups which focus on social interactions shows that a high incidence (13.58%) of these interactions occurred when bulls were housed. As these bulls were in a confined environment, there would have been competition for feed space or preferential lying space. Thus, an increase in displacements, and forcing other animals to stand in order to gain access to feed, water, or lying space would be expected [50]. However, Kenny and Tarrant [51] outlined that agonistic interactions were less intense within a confined space. Therefore, it could be assumed that although agonistic interactions at grass were less frequent, they may have been more intense.

The rumen temperatures observed during periods of agonistic or sexual behaviour show a less pronounced rise than would have been expected [24], particularly as animals are considered to be raised or excitable during such times. One potential reason for the observed results is that the bulls had not reached full sexual maturity at the time of observation; thus, these interactions may have been less intense [52]. Furthermore, bulls were in a stable social group and had already established a social hierarchy at the time of observation. Research had shown that agonistic and sexual interactions are reduced within a stable social group [53]. Figure 3 shows that there was a degree of variation between agonistic rumen temperatures of the three diets. That of the grazed bulls was particularly low; access to the drinker was the only source of competition for these bulls; thus it could be suggested that agonistic behaviours occurred around the time of drinking. Thus, rumen temperature may not have fully recovered from the rapid decline associated with the intake of water [10]. Conversely, agonistic interactions from the other two dietary groups are likely to have occurred around the time of consuming concentrates, which may further account for the observed differences.

The negative relationship between rumen temperature and step count is contrary to what would have been expected based on the findings of Gordon [54]. However, as further confirmed by the results in Figure 2, water intake causes a significant decline in rumen temperature [10,11]. Therefore, the low values in Figure 4 indicate that drinking is occurring during periods of both high and low activity. Figure 4 also shows a considerable proportion of temperatures are >40 °C when step count is <100. This is further supported by the results shown in Figure 3, where bulls had a significantly greater rumen temperature when stationary compared to that during locomotion at grass. A negative relationship between rumination time and activity in dairy cows has been well documented [55,56]. Thus, these elevated rumen temperatures during predominately stationary periods are most likely caused by rumination leading to additional heat production from rumen fermentation. Alternatively, behavioural changes are commonly associated with ill-health, with animals displaying lethargic behaviour, during which time they may exhibit a fever [57]. However, as no clinical signs of disease were observed during this study, it is unlikely that ill-health had any impact on rumen temperature.

## 5. Conclusions

In conclusion, diet had no consistent effect on rumen temperature. Significant differences in rumen temperature were observed between behaviours of young bulls. However, the results show that it is difficult to differentiate between behavioural interactions based purely on rumen temperature. All temperatures observed were within the normal range for healthy cattle. Therefore, there is limited need to take behaviour into account when using rumen temperature for the detection of ill-health in cattle.

## Figures and Tables

**Table 1:** Ethogram for behaviour observations in Study 1 and 2.

Rank	Behaviour Group	Behaviour	Description
**1**	Drinking	Drinking	Drinking
**2**	Agonistic	Bunt	Lowers its head, then uses the head to sharply strike another animal
		Head to head pushing	Pushes its head against the head of another individual
		Fight	Continued forceful head to head pushing, results in animals pushing each other off-balance or across the ground
		Pushing at the feed face ^y^	Pushing another animal at the feed face in order to gain access to feed
**3**	Sexual	Mount intention	Head and shoulders are raised, and weight is shifted to the rear, at least one front hoof remains on the ground
		Attempt to mount	Both front feet simultaneously leave the ground, but the animal does not become positioned on the mountee’s body
		Mounting	Lifts its forelegs off the ground and rests the chest on the body of another animal
		Sucking	Cross-sucking /drinking urine from another animal
**4**	Agonistic or sexual recipient	Avoids slowly	Moves away to avoid the aggressor slowly, does not turn towards the aggressor
		Moves away with speed	Moves away from the aggressor quickly
		Retaliates	Retaliates with an attack (bunt or push) towards the aggressor. No more than two physical responses
		Fights	Retaliates with continued aggressive behaviour. Behaviour is then scored based on agonistic behaviour group
**5**	Affiliative	Licking	Licking another animal
		Sniffing	Sniffing another animal
		Touching/rubbing	Touching or rubbing its head against the body of another animal
		Grooming/scratching	Animal grooming itself or scratching on bars in the pen
**6**	Affiliative recipient	Recipient of an affiliative behaviour	Being sniffed, touched or licked by another animal
**7**	Concentrates	Eating concentrates	Eating concentrates
**8**	Locomotion	Out of pen ^y^	Cattle out of pen to be weighed
		Walking	Walking
		Trotting	Trotting
**9**	Forage	Cantering ^x^	Cantering
		Grazing ^x^	Eating grass
		Eating silage ^y^	Eating silage
**10**	Stationary	Environmental exploring	Standing while sniffing, liking and biting an object in the environment
		Standing	Standing—appears to be doing nothing
		Lying	Lying down—either sleeping or resting

Rank is based on the priority level each behaviour group was considered when condensing behaviours into five-minute intervals; ^x^ = behaviours that were only scored in Study 1; ^y^ = behaviours that were only scored in Study 2.

**Table 2:** Chemical composition of feedstuffs.

Study	1:At Grass		2: Housed	
Feedstuff	Grass	Concentrates	Grass Silage	Concentrates
DM (g/kg F)	128	946	281	949
CP (g/kg DM)	200.0	-	-	-
Water soluble sugars (g/kg DM)	69.6	-	-	-
ME (MJ/kg DM)	11.0	-	-	-
ADF (g/kg DM)	299.0	114.6	316.4	130.5
NDF (g/kg DM)	-	308.2	541.2	298.0
Ash (g/kg DM)	-	63.8	113.0	83.2
Nitrogen (g/kg DM)	-	29.5	18.8	28.2

DM, dry matter. CP, crude protein. ME, metabolisable energy. ADF, acid detergent fibre. NDF, neutral detergent fi bre The chemical composition of grass was determined via NIRS analysis, and that of grass silage and concentrates was determined by wet chemistry analysis.

**Table 3:** Incidence rate (%) of behaviours during Study 1 and Study 2.

Behaviour Group	1: At Grass		2: Housed	
	IR (%)	SED	IR (%)	SED
Drinking	3.45	0.261	3.86	0.626
Agonistic	0.80	0.093	2.61	0.595
Sexual	0.83	0.103	3.20	0.700
Agonistic or sexual recipient	0.18	0.027	0.92	0.186
Affiliative	0.94	0.100	5.84	0.569
Affiliative recipient	0.72	0.088	1.01	0.140
Stationary	52.25	0.859	65.11	1.809
Locomotion	0.46	0.034	1.66	0.343
Concentrates	-	-	6.19	0.488
Foraging	-	-	10.91	0.611
Feeding *	40.63	0.896	-	-

* Concentrates and foraging have been grouped together as the grazed treatment did not have access to concentrates. IR, incidence rate.

**Table 4:** Incidence rate (%) of behaviours according to diet during Study 1 and Study 2.

Behaviour Group	Study 1: At Grass					Study 2: Housed					
	Grazed	Grazed + 2 kg	Grazed + ad Lib	SED	*p*-Value	2 kg conc.	4 kg conc.	ad Lib conc	Housed + ad Lib	SED	*p*-Value
Drinking	2.63	3.93	3.77	0.639	ns	1.58	2.40	6.09	5.36	1.770	ns
Agonistic	0.57	0.70	1.13	0.229	ns	2.69	4.38	2.25	1.12	1.682	ns
Sexual	0.81	0.70	0.98	0.253	ns	1.39	3.16	3.32	4.92	1.980	ns
Agonistic or sexual recipient	0.15	0.19	0.19	0.065	ns	0.45	0.85	1.12	1.25	0.520	ns
Affiliative	0.88	0.69	1.24	0.244	ns	2.95 ^a^	4.00 ^a^	3.94 ^a^	12.47 ^b^	1.610	<0.001
Affiliative recipient	0.57	0.55	1.02	0.216	ns	1.51	1.28	0.40	0.85	0.395	ns
Stationary	42.94 ^a^	46.75 ^a^	67.05 ^b^	2.105	<0.001	68.17	63.69	67.89	60.69	5.115	ns
Locomotion	0.23 ^a^	0.63 ^b^	0.51 ^b^	0.083	<0.001	2.22	2.48	0.65	1.29	0.969	ns
Concentrates	-	-	-	-	-	3.04 ^a^	5.34 ^ab^	8.40 ^b^	7.97 ^b^	1.381	<0.05
Foraging	-	-	-	-	-	17.43 ^b^	13.26 ^b^	7.58 ^a^	5.38 ^a^	1.726	<0.01
Feeding *	51.61 ^c^	46.05 ^b^	24.24 ^a^	2.196	<0.001	-	-	-	-	-	-

* Concentrates and foraging have been grouped together as the grazed treatment did not have access to concentrates. a-b represent significant differences between diets in each study.
